# High-Voltage Electrical Discharge Extraction of Polyphenols from Winter Savory (*Satureja montana* L.): Antioxidant Assessment and Chemometric Interpretation

**DOI:** 10.3390/plants14142214

**Published:** 2025-07-17

**Authors:** Kristian Pastor, Nataša Nastić, Aleksandra Gavarić, Siniša Simić, Ante Lončarić, Marija Banožić, Krunoslav Aladić, Stela Jokić, Jelena Vladić

**Affiliations:** 1 Faculty of Technology, University of Novi Sad, Bulevar cara Lazara 1, 21000 Novi Sad, Serbia; 2 Faculty of Food Technology Osijek, Josip Juraj Strossmayer University of Osijek, Franje Kuhača 20, 31000 Osijek, Croatiastela.jokic@ptfos.hr (S.J.); 3 Faculty of Agriculture and Food Technology, University of Mostar, Biskupa Čule bb, 88000 Mostar, Bosnia and Herzegovina; 4 REQUIMTE, NOVA School of Science and Technology, NOVA University, Largo da Torre, 2829-516 Caparica, Portugal

**Keywords:** high-voltage electrical discharge, winter savory, polyphenolics, green extraction, antioxidant activity

## Abstract

This study investigated the potential of high-voltage electrical discharge (HVED), as a green, non-thermal extraction technology, for recovering polyphenols from winter savory (*Satureja montana* L.). Key process parameters, including frequency (40, 70, 100 Hz) and extraction time (1, 5, 15, 30, 45 min), were optimized, using water as a solvent and maintaining a constant solid-to-liquid ratio of 1:100 g/mL. The extracts were characterized for total polyphenol content (TPC), total flavonoid content (TFC), and antioxidant activity (DPPH, ABTS, FRAP), while individual phenolics were quantified via HPLC-DAD. Multivariate chemometric analyses, including Pearson correlation, heatmap clustering, and principal component analysis (PCA), were employed to reveal relationships between extraction conditions, polyphenolic profiles, and antioxidant activities. The results showed strong correlations between TPC, TFC, and antioxidant activity, with compounds such as quercetin-3-D-galactoside, procyanidin A2, and rutin identified as key contributors. Among the tested conditions, extraction at 70 Hz for 45 min provided the highest polyphenol yield and bioactivity. The application of HVED demonstrated its potential as an efficient and environmentally friendly technique for obtaining phenolic-rich extracts. In addition, the use of chemometric tools provided useful insights for optimizing extraction conditions and understanding the contributions of specific compounds to bioactivity. These results support future applications in clean-label product development and contribute to broader efforts in sustainable ingredient production for the food, cosmetic, and nutraceutical sectors.

## 1. Introduction

*Satureja montana* L., commonly known as winter savory, is a perennial herbaceous plant belonging to the Lamiaceae family. This medicinal aromatic plant is versatile and known for its culinary, medicinal, and aromatic properties; therefore, it is an important species in both traditional and modern herbal practices. Winter savory is particularly rich in essential oils, with carvacrol and thymol as primary components, which has drawn significant research interest. Essential oil extraction methods such as hydrodistillation, Soxhlet extraction, and maceration with organic solvents have been widely studied [1,2,3]. Advanced extraction techniques have also been investigated. Djordjevic et al., for instance, examined hydrodistillation, solvent-free microwave extraction, and microwave extraction with a Clevenger apparatus to analyze oil composition in winter savory [2]. Additionally, green solvents such as supercritical fluids and subcritical water extractions have enabled the efficient isolation of monoterpenes like carvacrol, thymoquinone, camphor, borneol, caryophyllene, and *p*-cymene from winter savory [4,5,6].

Beyond essential oils, winter savory is also a valuable source of polyphenolic compounds, which significantly contribute to its pharmacological potential. While research on the polyphenols of winter savory is less extensive, studies have revealed a range of biologically important phenolic acids and flavonoids. However, most studies on phenols in winter savory have used conventional extraction methods and toxic organic solvents to obtain extracts. Zeljković et al. produced phenolic-rich extracts containing vanillic acid, syringic acid, ferulic acid, sinapic acid, and rosmarinic acid via continuous Soxhlet extraction with methanol and chloroform, followed by a liquid–liquid extraction clean-up (parts of each extract were chemically modified with diazomethane) [7]. Additionally, Cetkovic et al. identified various phenolic acids and flavanols, including protocatechuic acid, catechin, vanillic acid, caffeic acid, syringic acid, epicatechin, *p*-coumaric acid, and ferulic acid, through successive extractions with organic solvents such as methanol, petroleum ether, chloroform, ethyl acetate, and n-butanol [1].

In extracts from *S. montana* subsp. *kitaibelii* (Wierzb. ex Heuff.) P.W. Ball obtained through conventional extraction with methanol, ethanol, and acetone, chlorogenic acid was identified as the most abundant phenolic acid [8]. Tinctures prepared from winter savory by maceration or remaceration were rich in polyphenols, including caffeic, rosmarinic, and chlorogenic acids, as well as (-)-catechin and rutin, identified using the HPTLC method [3]. Rosmarinic acid was the primary phenolic compound in winter savory decoctions prepared by boiling in distilled water, along with other derivatives such as salvianolic and lithospermic acids. Salvianolic acid A and possibly lithospermic acid A were also identified. Luteolin, apigenin, and quercetin were present as major aglycones, with derivatives like luteolin-*O*-di-glucuronide and quercetin-*O*-glucuronide detected in winter savory [9]. Additionally, eight phenolic compounds, including rutin, quercetin, caffeic acid, *p*-coumaric acid, ellagic acid, protocatechuic acid, rosmarinic acid, and syringic acid, were quantified using HPLC in methanolic and ethanolic extracts [10].

Green techniques have been shown to produce winter savory extracts rich in phenols, although phenolic content in these studies was mainly analyzed by spectrophotometric methods without detailed identification of individual phenolic components. For instance, Zekovic et al. used microwave-assisted extraction to produce polyphenol-rich extracts from winter savory [11]. Subcritical water was also effective for obtaining phenol- and flavonoid-rich extracts [5], while Acimovic et al. assessed the total phenolic content of extracts obtained through ultrasound, microwave, and subcritical water-assisted extractions [12]. Individual phenolic components have been studied in extracts obtained with alternative solvents, deep eutectic systems, which were suitable for extracting rutin and rosmarinic acid from winter savory [13].

Developing green technologies and protocols for extracting compounds from potent natural resources like winter savory reduces reliance on toxic solvents, minimizes environmental impact, and enhances resource efficiency. This approach makes extraction processes more sustainable and economically viable for industrial applications. It also ensures that valuable compounds are extracted with a minimal ecological footprint, expanding the potential of traditional natural resources in industries such as pharmaceuticals, cosmetics, and food. Sustainable extraction protocols allow plants like winter savory to meet societal needs for natural, eco-friendly products while supporting biodiversity, underscoring the clear need for eco-friendly extraction methods.

One promising green technology, High-Voltage Electric Discharge (HVED), has recently advanced due to its cost-effectiveness, low operating temperatures, and reduced extraction times [14]. HVED concentrates high levels of energy within an aqueous suspension placed between a high-voltage needle electrode and a plated/grounded electrode. A rapid electrical discharge within the plasma channel triggers an electron avalanche and secondary electrohydrodynamic phenomena, such as shock waves, which lead to cell membrane electroporation and mechanical disintegration of cell structures [15]. HVED has demonstrated effectiveness for extracting phenols from various plant sources, including winery wastes [16] and dill seed [17].

While HVED has shown promise in extracting phenolic compounds from various plant materials, its application to *S. montana* has not yet been explored. Given the limited data on polyphenol extraction from this species using green technologies, this study aimed to evaluate HVED as a sustainable method for obtaining polyphenol-rich extracts. The effects of different HVED parameters on extraction efficiency, phenolic composition, and antioxidant activity were assessed to identify conditions suitable for efficient and environmentally friendly extraction.

## 2. Results and Discussion

### 2.1. Effect of HVED Parameters on TPC and TFC of Winter Savory Extracts

**Figure 1:**
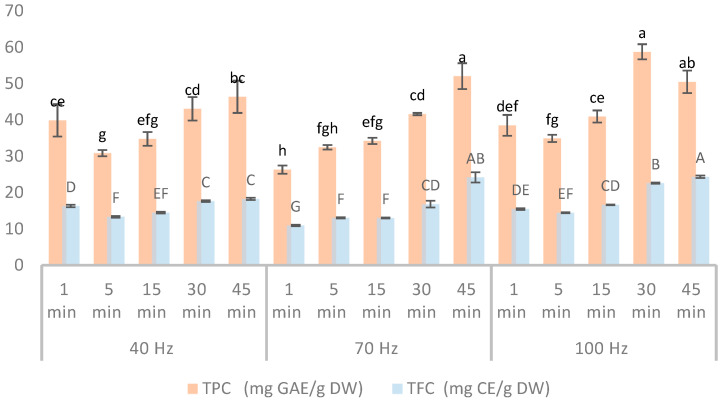
TPC and TFC of wintery savory extracts obtained by HVED under different conditions. Different letters on the bars in the figures represent significant differences among the samples for each response, determined at a significance level of *p* < 0.05.

The results of this study demonstrate that both the TPC and TFC in winter savory extracts were significantly influenced by the HVED extraction parameters. The TPC of winter savory extracts exhibited a range from 26.32 ± 1.12 to 58.74 ± 2.08 mg GAE/g DW, while the amounts of total flavonoids in winter savory extracts varied from 10.93 ± 0.21 to 24.35 ± 0.37 mg CE/g DW (Figure 1, Table S1).

Among the tested conditions, the maximum phenolic yield (58.74 ± 2.08 mg GAE/g DW) was obtained under extraction parameters of 100 Hz frequency and a treatment duration of 30 min, followed closely by 45 min at the same frequency (50.47 ± 2.03 mg GAE/g DW). Similarly, the maximum TFC (24.35 ± 0.37 mg CE/g DW) was recorded at 100 Hz for 45 min, suggesting that both extended treatment time and higher frequency favor the release of flavonoid compounds. While HVED extraction at 100 Hz for 30 and 45 min yielded the highest phenolic and flavonoid contents, some lower frequency and shorter time combinations (e.g., 40 Hz, 30 min for TPC: 43.06 ± 1.75 mg GAE/g DW) also demonstrated relatively high extraction efficiency.

A clear trend was observed with higher frequencies and treatment times generally correlated with higher yields of both phenolic and flavonoid compounds. For instance, at 40 Hz, TPC increased by 35% from 5 to 45 min, while at 70 Hz, the TPC increase was twice as much (from 1 to 45 min). Similarly, TFC at 70 Hz increased from 10.93 mg CE/g DW (1 min) to 24.17 mg CE/g DW (45 min). Previous studies reported a positive effect of HVED frequency on TPC and TFC, where its maximal yield was achieved using the frequency of 100 Hz [18]. The high electrical discharges generated during HVED create transient pores in cellular membranes through electroporation, facilitating the mass transfer of phenolic and flavonoid compounds into the solvent. Furthermore, the positive effect of extraction time on the TPC was previously confirmed by Dukić et al., who reported the highest phenolic content from sugar beet waste at a maximum treatment time of 9 min [19]. Although the highest extraction time reported in their study is notably shorter than the one employed in the present investigation, the observed trend consistently highlights the importance of extraction time as an important variable in the recovery of phenolic compounds. A longer HVED treatment positively influences extraction yield by increasing the total energy input into the system, thereby enhancing the probability of disrupting the bonds between phenolic compounds and the cell wall matrix.

The TPC and TFC values from this study are consistent with the values reported for winter savory extracts obtained by ultrasound-assisted extraction employing 30% ethanol at different extraction times and ratios [20]. Similarly, comparable TPC values were observed in extracts obtained through conventional maceration performed over a 30 min period using 50% ethanol [21]. However, it is noteworthy that the TFC values reported in the current study were more than two-fold higher than those achieved by maceration, indicating a significantly improved extraction efficiency for flavonoid compounds. The increase in TFC is likely due not only to the improved permeability but also to the liberation of flavonoid compounds that are otherwise bound within vacuolar compartments of the cell wall matrix [16]. Subcritical water extraction of winter savory has been shown to yield higher TPC values at optimal conditions (200 °C and 20.8 min), as reported by Vladić et al. [5]. Even though this technique yielded high concentrations of total phenolics, the overall profile may be affected, and some valuable compounds may be lost or transformed into less active forms due to the significant risk of thermal degradation for many heat-sensitive compounds. Microwave-assisted extraction of winter savory also yielded comparable or higher TPC values ranging from 29.5 to 74.4 mg GAE/g DW and TFC values between 24 and 48 mg CE/g DW, depending on the solvent system, time, and irradiation power [11]. However, among all tested parameter combinations in this study, only the use of 50% ethanol as a solvent consistently resulted in higher TPC values, regardless of time and power. This difference can be attributed to the polarity and solvent properties of 50% ethanol, which is particularly efficient in solubilizing a wide range of phenolic compounds, which may not be extracted as efficiently with either pure ethanol or water.

### 2.2. Effect of HVED Parameters on Antioxidant Activity of Winter Savory Extracts

**Figure 2:**
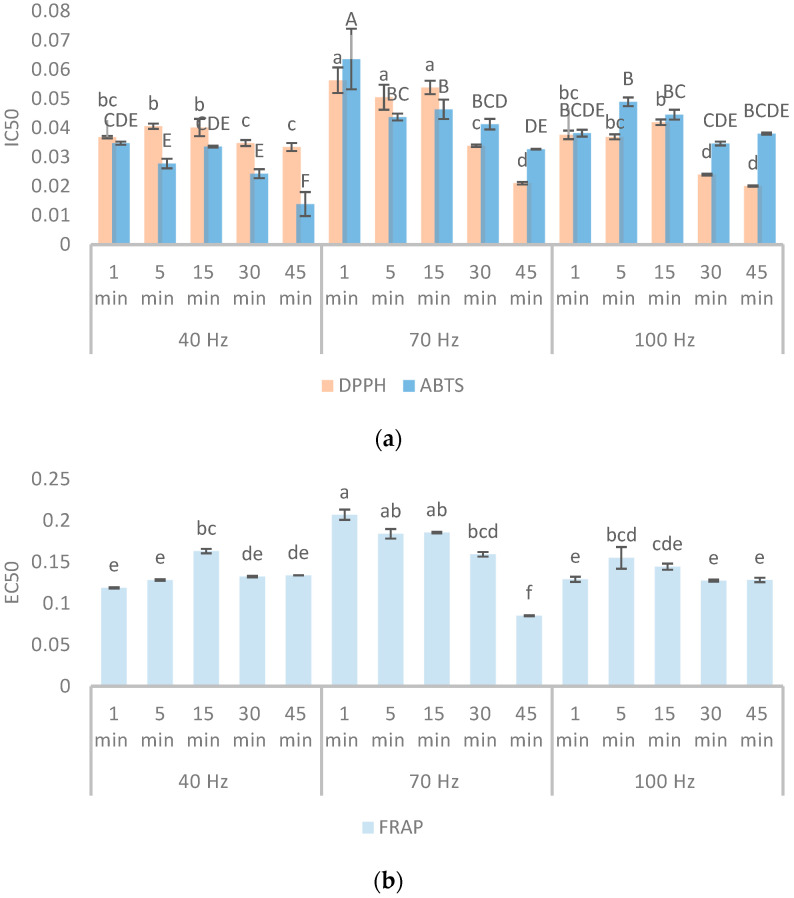
Antioxidant activities (mg/mL) of wintery savory extracts obtained by HVED at different conditions: (**a**) DPPH and ABTS; (**b**) FRAP. Different letters on the bars in the figures represent significant differences among the samples for each response, determined at a significance level of *p* < 0.05.

The in vitro antioxidant activity of the extracts was evaluated using three different assays: two based on free radical scavenging (DPPH and ABTS) and one assessing the ferric ion (Fe^3+^) reducing capacity (FRAP). The results obtained from all assays are presented in Figure 2 (Table S2).

The antioxidant activity assayed by DPPH showed that the HVED extract of winter savory obtained using 100 Hz frequency for 45 min displayed the highest radical scavenging activity (IC_50_ = 0.020 mg/mL), while the extract obtained by HVED at 70 Hz for 1 min showed the lowest (IC_50_ = 0.056 mg/mL) radical scavenging capacity. The strongest antioxidant activity was followed closely by 70 Hz for 45 min (IC_50_ = 0.021 mg/mL) and 100 Hz for 30 min (IC_50_ = 0.024 mg/mL). This suggests that longer extraction times at higher frequencies are particularly effective at enhancing the radical scavenging ability of winter savory extracts. These results are consistent with a study by Nastić et al., who found that higher HVED frequencies enhance the extraction of antioxidant compounds through intensified electroporation and cell wall disruption [18]. The prolonged exposure likely increases the diffusion of bioactive compounds into the solvent, avoiding thermal or oxidative degradation. The values of the DPPH found for the winter savory were lower (higher antioxidant activity) than those reported by Čutović et al. for the extracts prepared with 30% ethanol using ultrasound-assisted extraction [20]. The observed IC_50_ values for winter savory ranged from 2.75 to 3.93 mg/mL, depending on extraction time and solvent-to-solid ratio. In another study, subcritical water extraction achieved lower IC_50_ values at high temperatures [5]. However, using high-temperature conditions in subcritical water extraction leads to the formation of various thermochemical conversion products [21]. These newly formed compounds, which include Maillard reaction products, can significantly influence the overall bioactivity of the extract. Although these reactions can improve specific functional properties, they may also modify the effects of the native phytochemicals, making it difficult to determine how much of the observed bioactivity is due to the original plant compounds versus newly formed substances.

The ABTS activity of the winter savory extracts ranged from 0.014 to 0.063 mg/mL. Among the HVED extracts, the extract obtained at 40 Hz for 45 min showed the highest ABTS radical scavenging activity. Antioxidant activity decreased at higher frequencies and shorter durations, as seen in 70 Hz for 1 min (IC_50_ = 0.064 mL/mL) and 100 Hz for 5 min (IC_50_ = 0.049 mL/mL). This is in accordance with previous observations that HVED can exert dual effects: promoting compound release through electroporation while simultaneously inducing degradation via radical formation or localized heating [22]. The difference between optimal conditions for DPPH and ABTS assays could reflect differences in antioxidant polarity. DPPH radicals are more reactive with hydrophobic antioxidants, while ABTS can interact with both hydrophilic and lipophilic compounds [23].

The FRAP of winter savory extracts ranged from 0.066 to 0.207 mg/mL, with the maximum antioxidant activity measured in HVED extract at 70 Hz for 45 min being in line with DPPH results, which also showed a strong antioxidant response under similar conditions. On the other side, FRAP values at short extraction times and lower frequencies (0.119 mg/mL at 40 Hz, 1 min) were higher, indicating reduced activity. These findings are in line with those of Žuntar et al., who noted that ferric-reducing power increases with higher phenolic content, which in turn is modulated by the intensity of HVED processing [24]. However, overly high initial FRAP values (0.207 mg/mL at 70 Hz, 1 min) may suggest incomplete release or possible interference from the plant matrix.

### 2.3. Polyphenolic Composition of HVED Extracts Analyzed by HPLC-DAD

HPLC-DAD was employed to characterize the major polyphenolic compounds in the HVED winter savory extracts, as detailed in Table 1 (Table S3). Fifteen phenolic compounds and their derivatives were identified and quantified, including phenolic acids (gallic, ferulic, chlorogenic, caffeic, and *p*-coumaric), flavan-3-ols (epigallocatechin, catechin, epicatechin), procyanidins (A2, B1, B2), and flavonols and their glycosides (myricetin, rutin, quercetin, and quercetin-3-*O*-galactoside).

As can be seen in Table 1, the winter savory extracts were particularly rich in myricetin (646.05 µg/g DW), followed by epigallocatechin (384.69 µg/g DW), procyanidin B1 (367.20 µg/g DW), rutin (298.40 µg/g DW), and procyanidin B2 (199.50 µg/g DW). So far, myricetin has been shown to possess a wide range of biological activities and therapeutic effects against various diseases, including multiple types of cancer, inflammatory conditions, thrombosis, cerebral ischemia, diabetes, Alzheimer’s disease, and infections caused by pathogenic microbes [25]. Its high concentration in winter savory extracts further reinforces the plant’s potential as a functional food ingredient with significant health-promoting properties. The significant content of flavan-3-ols suggests that winter savory may possess strong anti-radical and cardioprotective properties, similar to those attributed to green tea catechins. Rutin, a glycosylated derivative of quercetin, was also highly abundant in winter savory, which aligns with earlier reports where rutin has been identified as one of the major flavonoids in *Satureja* species [13,26]. Its hepatoprotective, renoprotective, and cardioprotective activities further enhance the value of winter savory in medicinal applications [27]. Winter savory was also rich in procyanidin B1 and B2, compounds known not only for their ability to neutralize free radicals and protect cells from oxidative stress, but also for their anti-inflammatory and antimicrobial properties against a range of bacterial and fungal pathogens [28]. The presence of other phenolic compounds, including caffeic acid, *p*-coumaric acid, ferulic acid, and flavanols (catechin and epicatechin) was previously reported in the winter savory macerates [1]. The co-presence of these compounds with phenolic compounds may contribute to synergistic antioxidant effects. In addition, the TPC values obtained using the Folin–Ciocalteu assay were higher than the sum of the individually quantified polyphenols. This discrepancy is commonly reported in the literature and can be explained by the non-specific nature of the Folin–Ciocalteu reagent, which reacts not only with polyphenols but also with other reducing compounds present in the extract. In addition, the HPLC-DAD analysis is limited to compounds that can be reliably identified and quantified using available reference standards, meaning that undetected or minor phenolics may also contribute to the total phenolic content.

HVED treatment at 100 Hz for 45 min yielded the highest content of phenolic compounds, particularly procyanidins, epigallocatechin, myricetin, and rutin. At the same frequency, a 30-min extraction yielded the highest concentrations of gallic, chlorogenic, and *p*-coumaric acids, while for quercetin, the optimal extraction time was 15 min. On the other hand, HVED at 40 Hz for 1 min gave the lowest extraction efficiency, especially for larger flavonoid structures like procyanidins and epigallocatechin, which were mostly absent. Caffeic acid, as the most dominant phenolic acid in HVED extracts, peaked at 100 Hz, 45 min, more than six times higher than at lower frequencies. This sharp increase suggests that prolonged HVED exposure facilitates the hydrolysis of bound esters and further liberates caffeic acid.

**Figure 3:**
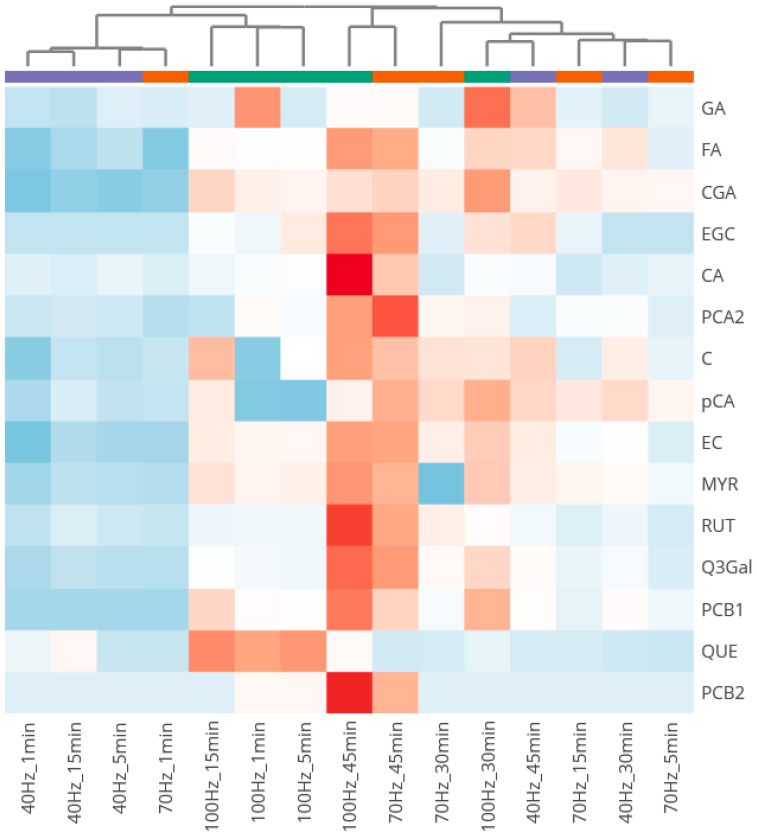
Heatmap clustering of HVED extract samples based on HPLC-DAD-detected bioactive compounds. Gallic acid (GA), ferulic acid (FA), chlorogenic acid (CGA), epigallocatechin (EGC), caffeic acid (CA), procyanidin A2 (PCA2), catechin (C), *p*-coumaric acid (pCA), epicatechin (EC), myricetin (MYR), rutin (RUT), quercetin-3-*O*-galactoside (Q3Gal), procyanidin B1 (PCB1), quercetin (QUE), and procyanidin B2 (PCB1). The heat map uses a blue-to-red color scale, where blue indicates lower concentrations and red represents higher concentrations.

Figure 3 shows the heatmap clustering of extract samples obtained by HVED under different extraction conditions, based on polyphenolic compounds detected by HPLC-DAD. High concentrations of specific compounds are represented in red, while low concentrations are indicated in blue.

The heatmap revealed distinct clustering patterns driven by HVED extraction parameters, frequency, and extraction time, emphasizing their influence on the phenolic and flavonoid composition of the extracts. Samples obtained at the lowest frequency of 40 Hz and short extraction times (1, 5, and 15 min), along with the sample extracted at 70 Hz for 1 min, exhibited similar compositions. These samples clustered on the left side of the heatmap dendrogram, showing low concentrations of all HPLC-identified components.

Two additional clusters were identified: the first comprised samples extracted at 100 Hz with short treatment times (1, 5, and 15 min), and the second included samples obtained at 40 Hz for longer extraction durations (30 and 45 min), 70 Hz for intermediate times (5 and 15 min), and 100 Hz for 30 min.

A distinct cluster was formed by extracts obtained with the longest treatment time (45 min) at higher frequencies (70 and 100 Hz), which exhibited the highest concentrations of detected compounds. Notably, these samples were particularly rich in procyanidin B2, caffeic acid, and rutin, followed by procyanidin B1, quercetin-3-*O*-galactoside, myricetin, epicatechin, catechin, procyanidin A2, epigallocatechin, and ferulic acid.

Thus, samples extracted at higher frequencies (e.g., 70 and 100 Hz) and longer treatment times (45 min) yielded significantly higher concentrations of bioactive compounds, likely contributing to greater antioxidant activity. Conversely, shorter treatment times and lower frequencies resulted in lower phenolic recovery, forming separate clusters indicative of suboptimal extraction conditions.

### 2.4. Correlation Analysis of Bioactive Compounds with TPC, TFC, and Antioxidant Activities

**Figure 4:**
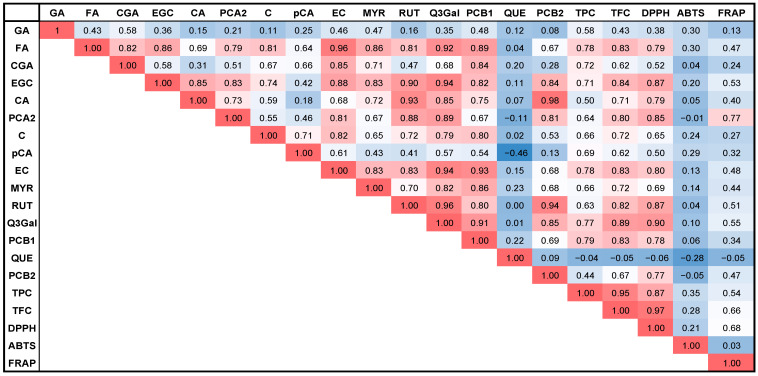
Pearson correlation analysis of HPLC-DAD-detected phenolic and flavonoid compounds, TPC, TFC, and antioxidant activities (DPPH, ABTS, and FRAP). Gallic acid (GA), ferulic acid (FA), chlorogenic acid (CGA), epigallocatechin (EGC), caffeic acid (CA), procyanidin A2 (PCA2), catechin (C), *p*-coumaric acid (pCA), epicatechin (EC), myricetin (MYR), rutin (RUT), quercetin-3-*O*-galactoside (Q3Gal), procyanidin B1 (PCB1), quercetin (QUE), and procyanidin B2 (PCB1).

Figure 4 presents the Pearson correlation analysis of eluting bioactive compounds, TPC, TFC, and antioxidant activities determined by DPPH, ABTS, and FRAP assays.

Strong positive correlations (r > 0.85) were observed between TPC, TFC, and antioxidant activity measured by DPPH, indicating that phenolic compounds significantly contribute to radical scavenging potential. Among individual bioactives, quercetin-3-*O*-galactoside (r = 0.90), rutin (r = 0.87), procyanidin A2 (r = 0.85), epigallocatechin (r = 0.87), and epicatechin (r = 0.80) showed the strongest correlations with DPPH activity, followed by ferulic acid and caffeic acid (r = 0.79), procyanidin B1 (r = 0.78), and procyanidin B2 (r = 0.77).

The highest correlation with antioxidant activity measured by FRAP was observed for procyanidin A2 (r = 0.77). Other bioactives exhibited moderate correlations with FRAP, including TFC (r = 0.66), quercetin-3-*O*-galactoside (r = 0.55), TPC (r = 0.54), epigallocatechin (r = 0.53), epicatechin (r = 0.48), and FA and PCB2 (r = 0.47). In contrast, correlations between ABTS activity and TPC (r = 0.35), gallic acid and ferulic acid (R = 0.30), *p*-coumaric acid (r = 0.29), and TFC (r = 0.28) were relatively low.

Negative correlations were consistently observed for quercetin across all antioxidant assays (DPPH, ABTS, FRAP) and total contents (TPC, TFC). However, strong positive correlations were recorded for quercetin-3-*O*-galactoside (r = 0.77–0.89), ferulic acid (r = 0.78–0.83), epigallocatechin (r = 0.71–0.84), epicatechin (r = 0.78–0.83), and procyanidin B1 (r = 0.77–0.89), confirming their significant roles in antioxidant potential.

**Figure 5:**
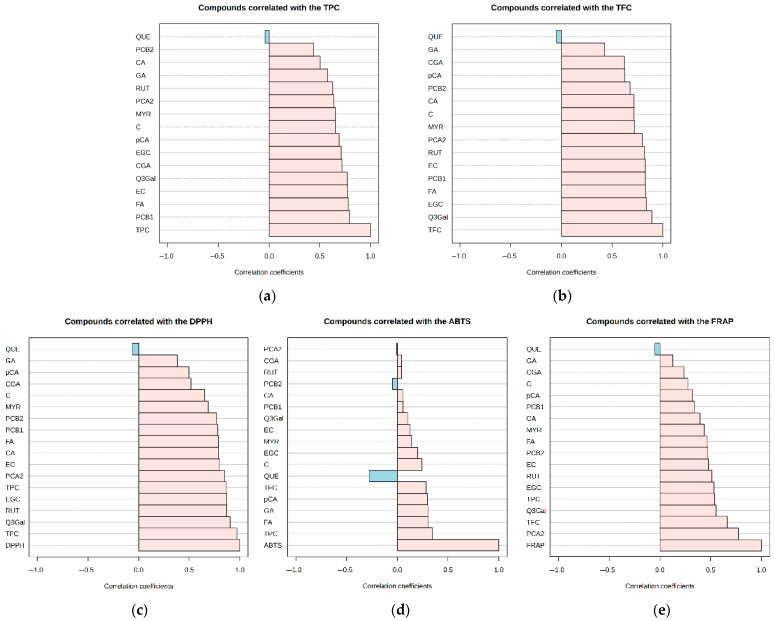
Correlation analysis of bioactive compounds with: (**a**) TPC, (**b**) TFC, (**c**) DPPH, (**d**) ABTS, and (**e**) FRAP antioxidant activities. Gallic acid (GA), ferulic acid (FA), chlorogenic acid (CGA), epigallocatechin (EGC), caffeic acid (CA), procyanidin A2 (PCA2), catechin (C), *p*-coumaric acid (pCA), epicatechin (EC), myricetin (MYR), rutin (RUT), quercetin-3-*O*-galactoside (Q3Gal), procyanidin B1 (PCB1), quercetin (QUE), and procyanidin B2 (PCB1).

This was further validated by correlating eluting bioactive compounds with specific variables, including TPC, TFC, and antioxidant activities determined via DPPH, ABTS, and FRAP assays, as illustrated in Figure 5.

Figure 5 further supports the negative correlation of quercetin with all variables studied. TPC and TFC were found to be the most strongly correlated with each other, followed by procyanidin B1, quercetin-3-*O*-galactoside, ferulic acid, epicatechin, and epigallocatechin in both cases. Other compounds exhibited weaker positive correlations with the total phenolic and flavonoid contents.

When DPPH was used as the reference variable, TFC was identified as the primary contributor to antioxidant activity, followed by the compounds: quercetin-3-*O*-galactoside > rutin > epigallocatechin > TPC > procyanidin A2 > epicatechin > caffeic acid > ferulic acid > procyanidin B1 > procyanidin B2 > myricetin > chlorogenic acid > *p*-coumaric acid > gallic acid.

For the ABTS assay, TPC emerged as the key contributor to antioxidant activity, with the following ranking: ferulic acid > gallic acid > *p*-coumaric acid > TFC > catechin > epigallocatechin > myricetin > epicatechin > quercetin-3-*O*-galactoside > procyanidin B1 > caffeic acid > rutin > chlorogenic acid > procyanidin B2. Procyanidin B2 exhibited a negative correlation with ABTS activity, together with quercetin.

In the case of the FRAP assay, procyanidin B2 was the most influential contributor to antioxidant activity, followed by the bioactives: TFC > quercetin-3-*O*-galactoside > TPC > epigallocatechin > rutin > epicatechin > procyanidin B2 > ferulic acid > myricetin > caffeic acid > procyanidin B1 > *p*-coumaric acid > catechin > chlorogenic acid > gallic acid.

Thus, such ranking the contributions of individual bioactive compounds to antioxidant activities (DPPH, ABTS, FRAP) and total contents of polyphenols and flavonoids provides a clear and interpretable overview of which compounds are most significant.

### 2.5. Principal Component Analysis (PCA) of HVED Extract Profiles

**Figure 6:**
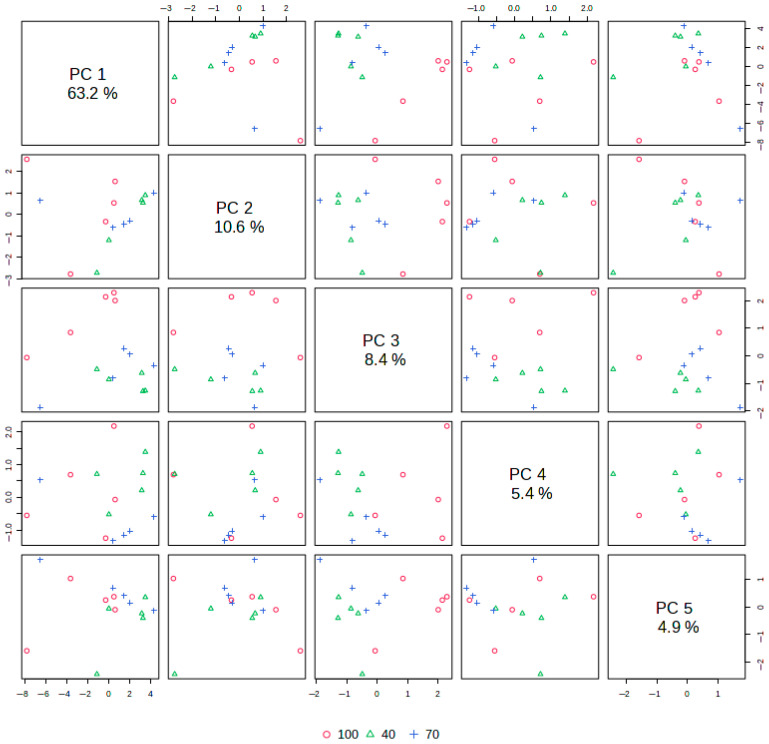
Principal component pairs and total variance explained by the first five components.

The PCA revealed that the first five principal components (PC1–PC5) accounted for 92.5% of the total variance, effectively separating samples based on HVED extraction conditions and their compound profiles, as shown in Figure 6.

**Figure 7:**
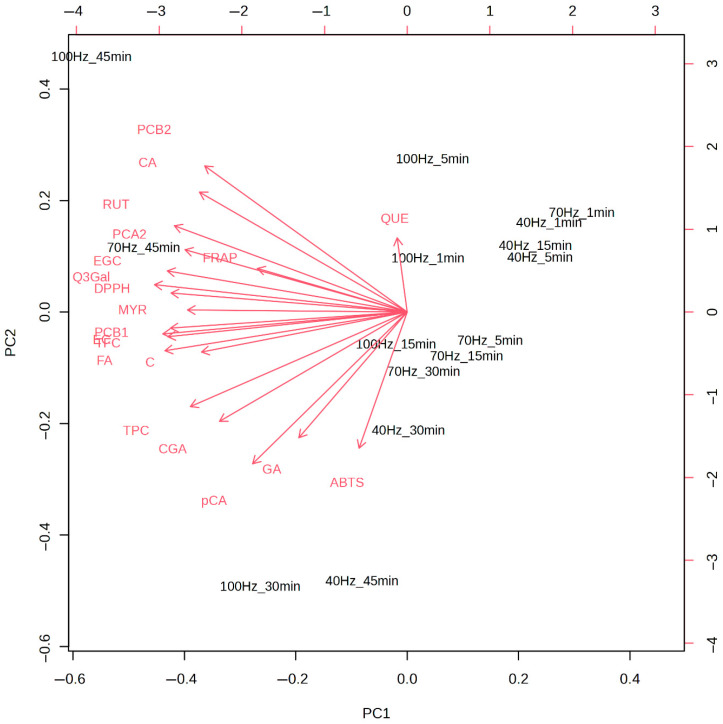
PCA bi-plot of extract samples obtained via HVED under different extraction conditions, representing scores (samples) and loadings (HPLC-DAD bioactives, TPC, TFC, and antioxidant activities: DPPH, ABTS, FRAP). Gallic acid (GA), ferulic acid (FA), chlorogenic acid (CGA), epigallocatechin (EGC), caffeic acid (CA), procyanidin A2 (PCA2), catechin (C), *p*-coumaric acid (pCA), epicatechin (EC), myricetin (MYR), rutin (RUT), quercetin-3-*O*-galactoside (Q3Gal), procyanidin B1 (PCB1), quercetin (QUE), and procyanidin B2 (PCB1).

Figure 7 presents the PCA bi-plot, illustrating extract samples obtained under various HVED extraction conditions (frequencies and extraction times) as scores, with identified eluting compounds (HPLC-DAD), total phenolic and flavonoid contents, and antioxidant activities (DPPH, ABTS, FRAP) represented as loadings.

Similar to the heatmap dendrogram in Figure 3, the PCA bi-plot shows that extract samples obtained at low frequencies (40 Hz) and short extraction times (1, 5, and 15 min), as well as 70 Hz for 1 min, cluster on the right side of the plot. This area is devoid of significant loadings, indicating the low extraction potential of HVED under these conditions.

In contrast, the highest quercetin content was observed in samples extracted at 100 Hz with short times (1 and 5 min), while the strongest antioxidant potential, as indicated by ABTS activity, was observed in samples extracted at 40 Hz for longer times (30 and 45 min). Notably, the ABTS loading shows an inverse correlation with quercetin content.

Samples extracted under higher frequencies (100 Hz) and longer times (30 and 45 min) exhibited significant extraction potential, with variables such as gallic acid, p-coumaric acid, procyanidin B2, and caffeic acid strongly associated with these conditions. The most promising extraction conditions were observed at 70 Hz for 45 min, yielding the highest concentrations of bioactives detected by HPLC, including antioxidant activities determined by DPPH and FRAP.

In the PCA bi-plot, FRAP activity predominantly correlated with procyanidin A2, rutin, and epigallocatechin, while DPPH activity showed stronger associations with quercetin-3-*O*-galactoside, epigallocatechin, and myricetin. Other bioactives, such as procyanidin B1, TFC, ferulic acid, catechin, TPC, chlorogenic acid, caffeic acid, and procyanidin B2, also contributed significantly to the antioxidant potential.

Pearson correlation analysis shows that procyanidin A2 and quercetin-3-*O*-galactoside have high r values with DPPH and FRAP, and in the PCA bi-plot, procyanidin A2 and quercetin-3-*O*-galactoside loadings closely correlate with DPPH and FRAP loadings, indicating they drive antioxidant activities. Furthermore, similar to the clustering observed in Figure 3, the sample with high bioactive content extracted at 70 Hz for 45 min is positioned in the PCA bi-plot near the loadings for most bioactive compounds and, thus, corresponding antioxidant activities. Samples with low bioactive recovery (e.g., 40 Hz, 1 min) will appear distant from these loadings, reinforcing their poor extraction potential.

Therefore, the bi-plot revealed clear groupings of samples, with optimal HVED conditions forming distinct clusters linked to superior bioactivity, while suboptimal conditions were more dispersed and exhibited lower bioactive recovery.

## 3. Materials and Methods

### 3.1. Plant Material and Chemicals

The cultivated winter savory (*S. montana* L.) aerial parts were purchased from the Institute for Medicinal Plants Research “Dr. Josif Pancic”, Belgrade, Serbia. The air-dried aerial parts of winter savory were milled (TSM6A013B electric grinder Bosch, Gerlingen, Germany), and the mean particle size (0.32 ± 0.05 mm) was determined by the vibration sieves set (CISA, Cedaceria, Spain).

Methanol and ultrapure water (LC-MS grade) were provided by Scharlau (Chemie S.A., Barcelona, Spain), formic acid was sourced from Merck (Darmstadt, Germany), and deionized water was produced in the lab with a MilliQ gradient system (Millipore, Bedford, MA, USA). Folin–Ciocalteu reagent, (±)-catechin (CAS: 154-23-4, ≥99.0%), and 1,1-diphenyl-2-picryl-hydrazyl-hydrate (DPPH•) (CAS: 1707-75-1) were sourced from Sigma-Aldrich GmbH (Steinheim, Germany). Gallic acid (CAS: 149-91-7, ≥97%) was obtained from Sigma (St. Louis, MO, USA), while potassium persulfate (99% pure) came from Acros Organics (Geel, Belgium). Ferulic acid (CAS: 537-98-4, ≥98%), chlorogenic acid (CAS: 327-97-9, ≥95%), caffeic acid (CAS: 331-39-5, ≥98.0%), *p*-coumaric acid (CAS: 1261170-80-2, ≥99%), epigallocatechin (CAS: 989-51-5, ≥98%), catechin (CAS: 154-23-4, ≥99.0%), epicatechin (CAS: 490-46-0, ≥98%), procyanidin A2 (CAS: 41743-41-3, ≥99%), myricetin (CAS: 529-44-2, ≥96.0%), rutin (CAS: 153-18-4, 90-95%), quercetin-3-*O*-galactoside (CAS: 96862-01-0, ≥98%), procyanidin B1 (CAS: 20315-25-7, ≥90%), quercetin (CAS: 117-39-5, ≥95%), and procyanidin B2 (CAS: -49-8, ≥90%), were acquired from Sigma-Aldrich (Merck KGaA, Darmstadt, Germany).

### 3.2. High-Voltage Electrical Discharge (HVED)-Assisted Extraction

Batch HVED-assisted extraction was conducted at various frequencies (40, 70, and 100 Hz) and treatment times (1, 5, 15, 30, and 45 min) using custom-built equipment (Ingeniare CPTS1, Faculty of Food Technology Osijek, Osijek, Croatia). The setup featured a 30 kV high-voltage pulse generator, a magnetic stirrer, and a treatment chamber with a needle-plate electrode configuration. The chamber contained a sharpened stainless-steel needle (2.5 mm diameter) as the positive electrode and a stainless-steel disc (45 mm diameter) as the ground electrode, separated by a 5 mm gap. During HVED, electrical discharges were generated within the water-based solvent system, housed in a 600 mL chamber. Extractions were carried out with a solid-to-liquid ratio of 100 mL/g at room temperature (25 °C), using negative polarity to maximize discharge efficacy in low-conductivity water. Each extraction was performed in triplicate, and the resulting extracts were vacuum-filtered and stored at 4 °C in a dark environment until analysis.

### 3.3. Total Phenols and Flavonoids Content

The total phenolic content (TPC) in winter savory extracts was determined using the Folin–Ciocalteu method [29], with absorbance measured at 750 nm. TPC levels were expressed as milligrams of gallic acid equivalent per gram of dry weight (mg GAE/g DW). The total flavonoid content (TFC) was assessed using the aluminum chloride colorimetric assay [30], with catechin as the standard, and absorbance measured at 510 nm. TFC levels were expressed as milligrams of catechin equivalent per gram of dry weight (mg CE/g DW). All experiments were conducted in triplicate, with results presented as mean values.

### 3.4. HPLC Analysis

Polyphenols analysis was carried out using high-performance liquid chromatography on a Jasco LC Net II system, equipped with an AS-4150 autosampler, a PU-4180 pump, and an MD-4010 PDA detector. The system was managed by JASCO ChromNAV Version 2.01.00 software (JASCO International Co., Ltd., Tokyo, Japan). Column C18 Kinetex (150 mm × 4.6 mm, 2.6 μm; Phenomenex, Torrance, CA, USA) was used. The mobile phase consisted of solvent A (water with 1% formic acid) and solvent B (methanol with 1% formic acid), with a gradient program starting from 95% A to 80% in 10 min, from 80% to 70% in 5 min, from 70% to 50% in 5 min, from 50% to 0% in 5 min and isocratic for 10 min at a flow rate of 1 mL/min. Five μL of the sample was double-injected onto the column maintained at 50 °C. UV–Vis absorption spectra of both standards and samples were recorded between 190 and 600 nm.

Phenols were detected at 280 nm (catechin, y = 1496.9x − 31,807; R^2^ = 0.9999; epicatechin, y = 6591x − 2097.6; R^2^ = 0.9993; procyanidin A2, y = 271x + 28,509; R^2^ = 0.9999, B1, y = 3184.4x + 140,717; R^2^ = 0.9952; and B2, y = 4661.8x − 1572.8; R^2^ = 0.9998; epigallocatechin, y = 8486x + 13,533; R^2^ = 0.9992; gallic acid, y = 20,543x + 27,568; R^2^ = 0.9999), 320 nm (caffeic acid, y = 39,450x + 2 × 10^7^ R^2^ = 0.9995; chlorogenic acid, y = 16,186x − 891,959; R^2^ = 0.9999; ferulic acid y = 4265.8x + 280,378; R^2^ = 0.9999, and *p*-coumaric acid, y = 4004.7x + 724,691; R^2^ = 0.9999), 360 nm (myricetin, y = 4705.9x – 7 × 106; R^2^ = 0.9998; quercetin, y = 2937.1x − 911,531; R^2^ = 0.9976; rutin, y = 35,321x + 24,302; R^2^ = 0.9998 and quercetin-3-O-galactoside, y = 1279.5x + 189,066; R^2^ = 0.9999). Quantification has been performed by external standard calibration, and the calibration range for each phenolic standard was 0.1–100 µg/mL. Phenolic content was expressed in micrograms per gram of dry weight (µg/g DW).

### 3.5. Assessment of Antioxidant Activity

#### 3.5.1. DPPH Assay

The free radical scavenging activity of winter savory extracts was measured using a spectrophotometric method as described by Espín et al. [31]. Absorbance was recorded at 517 nm, and the radical scavenging capacity (RSC) was calculated using Equation (1). Antioxidant activity was expressed as the IC_50_ value (mg/mL), representing the extract concentration required to achieve 50% RSC.

%RSC = 100 − ((A_sample_ × 100))/A_control_ (1)

where A_sample_ is the absorbance of the sample solution and A_control_ is the absorbance of the control. All experiments were performed in triplicate.

#### 3.5.2. ABTS Assay

The antioxidant capacity was evaluated using the Trolox equivalent antioxidant capacity (TEAC) assay [32]. ABTS radical cations (ABTS•+) were generated by reacting a 7 mM ABTS stock solution with 2.45 mM potassium persulfate, with the mixture left in the dark at room temperature for 12–16 h. For analysis, the ABTS•+ solution was diluted with 5 mM phosphate-buffered saline (pH 7.4) to an absorbance of 0.70 ± 0.02 at 730 nm. A 0.01 mL aliquot of each sample was mixed with 4 mL of diluted ABTS•+ solution, and the absorbance decrease was measured at 734 nm after 30 min at 30 °C. A blank was prepared using water instead of the extract. ABTS•+ radical scavenging activity was expressed as IC_50_ values (mg/mL). All experiments were performed in triplicate.

#### 3.5.3. FRAP Assay

The reducing power of winter savory extracts was determined by the FRAP method [33]. Various concentrations of extract were mixed with 2.5 mL of 0.2 M phosphate buffer (pH 6.6) and 2.5 mL of 1% potassium ferricyanide (K_3_Fe(CN)_6_). The mixture was incubated for 20 min at 50 °C. After incubation, 2.5 mL of a 10% trichloroacetic acid solution was added, and the mixture was centrifuged (Tehtnica Železniki, Železniki, Slovenia) for 10 min at 3000 rpm. The supernatant (2.5 mL) was mixed with 2.5 mL of bi-distilled water and 0.5 mL of 0.1% FeCl_3_ solution. Absorbance was measured at 700 nm. Antioxidant activity was expressed as the EC_50_ value (mg/mL), representing the extract concentration required for 50% reduction of Fe^3+^ ions in the reaction mixture. All experiments were performed in triplicate.

### 3.6. Statistical Analysis

All experimental measurements were performed in triplicate, and results were expressed as mean values ± standard deviation (SD). Statistical and chemometric analyses were conducted using the MetaboAnalyst platform (https://www.metaboanalyst.ca, accessed on 12 May 2025). Prior to analysis, the dataset was auto-scaled to ensure equal weighting of all variables. Chemometric tools applied included heatmap clustering, Pearson correlation analysis, and principal component analysis (PCA). Heatmap clustering was employed to visualize relationships between extract samples and the phenolic components measured using HPLC-DAD. Pearson correlation analysis was used to identify significant associations between TPC, TFC, individual phenolic compounds, and antioxidant activity indicators. PCA was performed to identify patterns in the dataset, reduce dimensionality, and explain variance based on extraction parameters and measured features. For antioxidant tests (DPPH, ABTS, FRAP), reciprocal values were used, allowing higher values to indicate stronger antioxidant activity. Data visualization and clustering results were interpreted to identify optimal HVED extraction conditions.

## 4. Conclusions

The study confirms that HVED is an efficient and eco-friendly technology for polyphenols extraction from winter savory. Through optimization of process parameters, frequency, and extraction time, the research identified that HVED at 70 Hz for 45 min yielded extracts with the highest total polyphenol and flavonoid contents, as well as the strongest antioxidant activity. Fifteen phenolic compounds and their derivatives were identified and quantified, including phenolic acids, flavan-3-ols, procyanidins, and flavonols and their glycosides. The winter savory extracts were particularly rich in myricetin, followed by epigallocatechin, procyanidin B1, rutin, and procyanidin B2. HVED at higher frequencies and longer times yielded winter savory extracts with the richest and most diverse phenolic profiles. Strong correlations between key phenolics and antioxidant activities (DPPH, FRAP) highlight the potential of the HVED technique in producing bioactive-rich extracts for industrial applications. The multivariate analyses (heatmap clustering and PCA) validated the influence of HVED parameters on extraction profiles, offering a foundation for its scaling in green extraction technologies. Future studies could explore solvent modifications or hybrid techniques to further enhance its efficiency.

## Figures and Tables

**Table 1:** HPLC-DAD analysis of winter savory extracts obtained by HVED at different extraction conditions.

Sample		Content (µg/g DW)				
		Gallic Acid	Ferulic Acid	Epigallocatechin	Chlorogenic Acid	Caffeic Acid
**40 Hz**	1 min	8.25 ± 0.49 ^i^	3.80 ± 0.14 ^g^	n.d.	10.10 ± 0.56 ^f^	7.70 ± 0.42 ^fg^
	5 min	14.90 ± 0.42 ^eg^	8.35 ± 0.35 ^f^	n.d.	13.15 ± 1.06 ^ef^	10.20 ± 1.84 ^ef^
	15 min	7.25 ± 0.07 ^hi^	6.60 ± 0.00 ^f^	n.d.	16.10 ± 0.14 ^e^	5.40 ± 0.28 ^g^
	30 min	11.90 ± 0.14 ^gh^	17.70 ± 2.10 ^bc^	n.d.	58.40 ± 1.97 ^d^	7.75 ± 0.77 ^fg^
	45 min	44.50 ± 1.84 ^c^	19.55 ± 0.35 ^b^	194.40 ± 9.29 ^c^	59.65 ± 0.63 ^d^	15.10 ± 0.00 ^cd^
**70 Hz**	1 min	13.65 ± 1.20 ^eg^	3.55 ± 0.21 ^g^	n.d.	16.3 ± 0.42 ^e^	5.80 ± 1.70 ^g^
	5 min	17.30 ± 1.27 ^e^	11.55 ± 0.49 ^e^	n.d.	57.4 ± 5.93 ^d^	9.65 ± 0.07 ^ef^
	15 min	16.35 ± 1.20 ^ef^	15.45 ± 0.35 ^cd^	69.35 ± 0.92 ^fg^	64.65 ± 6.12 ^d^	2.15 ± 0.07 ^h^
	30 min	12.10 ± 0.85 ^fg^	13.80 ± 0.42 ^de^	58.45 ± 1.06 ^g^	62.85 ± 1.48 ^d^	2.35 ± 0.21 ^h^
	45 min	24.60 ± 3.11 ^d^	25.40 ± 1.97 ^a^	328.55 ± 6.90 ^b^	75.5 ± 4.81 ^b^	41.50 ± 1.98 ^b^
**100 Hz**	1 min	57.90 ± 1.41 ^b^	14.10 ± 0.00 ^de^	80.10 ± 5.23 ^ef^	60.8 ± 2.82 ^d^	15.40 ± 0.14 ^cd^
	5 min	12.75 ± 1.91 ^g^	14.60 ± 0.85 ^d^	156.45 ± 9.54 ^d^	58.55 ± 5.44 ^d^	16.25 ± 0.92 ^c^
	15 min	15.40 ± 0.57 ^eg^	14.90 ± 0.57 ^d^	98.35 ± 2.33 ^e^	73.9 ± 1.40 ^b^	12.30 ± 0.28 ^de^
	30 min	66.70 ± 4.24 ^a^	19.85 ± 1.20 ^b^	174.10 ± 9.23 ^d^	102.4 ± 3.68 ^a^	15.65 ± 0.64 ^c^
	45 min	24.40 ± 1.70 ^d^	27.10 ± 1.13 ^a^	384.60 ± 1.27 ^a^	69.1 ± 3.69 ^c^	94.90 ± 3.96 ^a^
		**Procyanidin A2**	**Catechin**	***p*** **-Coumaric acid**	**Epicatechin**	**Myricetin**
**40 Hz**	1 min	20.90 ± 1.56 ^ef^	n.d.	12.70 ± 0.57 ^g^	11.70 ± 3.11 ^g^	93.60 ± 10.60 ^h^
	5 min	22.55 ± 0.77 ^ef^	37.15 ± 2.48 ^j^	18.05 ± 0.77 ^f^	21.65 ± 2.19 ^f^	144.00 ± 9.61 ^h^
	15 min	23.75 ± 0.49 ^ef^	42.75 ± 1.34 ^ij^	24.20 ± 0.71 ^e^	24.10 ± 0.85 ^f^	154.25 ± 13.08 ^h^
	30 min	42.30 ± 3.68 ^d^	99.55 ± 2.01 ^ef^	50.15 ± 2.33 ^b^	44.70 ± 0.86 ^d^	328.45 ± 6.71 ^f^
	45 min	27.00 ± 3.54 ^e^	124.45 ± 2.76 ^cd^	51.05 ± 1.06 ^b^	52.15 ± 0.07 ^c^	370.80 ± 11.60 ^ef^
**70 Hz**	1 min	12.50 ± 1.41 ^g^	45.20 ± 5.37 ^ij^	18.55 ± 0.49 ^f^	21.35 ± 2.62 ^f^	133.00 ± 8.20 ^h^
	5 min	29.40 ± 0.00 ^e^	65.00 ± 2.54 ^gh^	39.25 ± 1.06 ^d^	35.05 ± 1.06 ^e^	279.80 ± 7.50 ^g^
	15 min	41.50 ± 1.84 ^d^	54.00 ± 4.95 ^hi^	44.50 ± 1.71 ^c^	42.65 ± 3.46 ^d^	342.05 ± 21.14 ^f^
	30 min	50.30 ± 0.56 ^c^	109.85 ± 9.92 ^de^	50.30 ± 1.71 ^b^	50.90 ± 2.83 ^c^	4.30 ± 0.57 ^i^
	45 min	134.55 ± 11.81 ^a^	140.30 ± 9.76 ^bc^	66.80 ± 4.10 ^a^	78.85 ± 3.18 ^a^	562.25 ± 12.52 ^b^
**100 Hz**	1 min	47.25 ± 2.61 ^cd^	n.d.	2.50 ± 0.00 ^h^	48.60 ± 0.14 ^cd^	345.90 ± 2.83 ^f^
	5 min	40.20 ± 1.13 ^d^	82.35 ± 6.29 ^fg^	1.80 ± 0.14 ^h^	47.85 ± 1.20 ^cd^	360.60 ± 16.77 ^ef^
	15 min	15.65 ± 0.35 ^fg^	145.65 ± 1.77 ^b^	43.70 ± 1.70 ^c^	52.35 ± 2.62 ^c^	407.35 ± 26.09 ^d^
	30 min	52.30 ± 3.82 ^c^	108.45 ± 1.76 ^de^	67.65 ± 4.60 ^a^	64.15 ± 4.60 ^b^	486.25 ± 38.96 ^c^
	45 min	102.80 ± 7.35 ^b^	169.95 ± 5.59 ^a^	40.85 ± 2.33 ^d^	80.50 ± 5.80 ^a^	646.05 ± 63.57 ^a^
		**Rutin**	**Quercetin-3-D-galactoside**	**Procyanidin B1**	**Quercetin**	**Procyanidin B2**
**40 Hz**	1 min	22.85 ± 0.63 ^h^	7.10 ± 0.28 ^g^	n.d.	8.25 ± 0.21 ^d^	n.d.
	5 min	35.35 ± 2.05 ^gh^	13.70 ± 0.99 ^g^	n.d.	1.00 ± 0.00 ^f^	n.d.
	15 min	47.70 ± 2.97 ^fg^	16.45 ± 1.77 ^g^	n.d.	13.90 ± 0.14 ^c^	n.d.
	30 min	65.45 ± 7.21 ^ef^	42.70 ± 1.13 ^e^	145.55 ± 7.31 ^d^	2.20 ± 0.12 ^f^	n.d.
	45 min	71.75 ± 0.49 ^de^	50.60 ± 0.42 ^d^	141.85 ± 8.60 ^d^	3.35 ± 1.20 ^ef^	n.d.
**70 Hz**	1 min	29.35 ± 2.33 ^g^	12.70 ± 0.85 ^g^	n.d.	1.20 ± 0.28 ^f^	n.d.
	5 min	42.55 ± 1.06 ^g^	28.10 ± 1.70 ^f^	115.35 ± 1.91 ^ef^	1.40 ± 0.23 ^f^	n.d.
	15 min	49.70 ± 0.85 ^fg^	37.30 ± 1.84 ^e^	101.80 ± 1.27 ^f^	3.35 ± 0.49 ^ef^	n.d.
	30 min	107.20 ± 4.38 ^c^	51.30 ± 1.55 ^d^	124.90 ± 10.89 ^e^	3.05 ± 0.07 ^ef^	n.d.
	45 min	204.00 ± 6.93 ^b^	115.10 ± 0.57 ^b^	220.25 ± 22.27 ^c^	2.80 ± 0.28 ^ef^	107.30 ± 2.83 ^b^
**100 Hz**	1 min	67.90 ± 0.71 ^ef^	41.80 ± 2.26 ^e^	144.25 ± 0.49 ^d^	36.05 ± 0.49 ^b^	39.50 ± 4.38 ^c^
	5 min	70.35 ± 4.60 ^de^	39.30 ± 4.95 ^e^	139.60 ± 7.96 ^d^	38.65 ± 5.72 ^ab^	39.00 ± 3.54 ^c^
	15 min	66.10 ± 3.82 ^ef^	46.40 ± 2.97 ^de^	215.65 ± 14.92 ^c^	40.95 ± 1.48 ^a^	n.d.
	30 min	88.15 ± 8.41 ^cd^	75.55 ± 6.29 ^c^	281.00 ± 8.63 ^b^	6.75 ± 1.20 ^d^	n.d.
	45 min	298.40 ± 20.65 ^a^	138.90 ± 9.86 ^a^	367.20 ± 1.98 ^a^	13.25 ± 0.91 ^c^	199.50 ± 14.42 ^a^

n.d.—not detected. Different letters within the column represents significant differences among the samples, determined at a significance level of *p* < 0.05.

## Data Availability

The original contributions presented in this study are included in the article/Supplementary Materials. Further inquiries can be directed to the corresponding author.

## Supplementary Materials

The following supporting information can be downloaded at: https://www.mdpi.com/article/10.3390/plants14142214/s1.

## Abbreviations

The following abbreviations are used in this manuscript:

ABTS	2,2’-Azino-bis(3-ethylbenzothiazoline-6-sulfonic acid
CA	Caffeic acid
C	Catechin
CGA	Chlorogenic acid
DPPH	1,1-Diphenyl-2-picryl-hydrazyl-hydrate
EC	Epicatechin
EGC	Epigallocatechin
FA	Ferulic acid
FRAP	Ferric reducing antioxidant power
GA	Gallic acid
HVED	High-voltage electric discharge
MYR	Myricetin
PCA	Principal component analysis
PCA2	Procyanidin A2
pCA	*p*-Coumaric acid
PCB1	Procyanidin 1
PCB2	Procyanidin 2
Q3Gal	Quercetin-3-D-galactoside
QUE	Quercetin
RUT	Rutin
TFC	Total flavonoid content
TPC	Total phenolic content
