# Supplementary material for: High-Voltage Electrical Discharge Extraction of Polyphenols from Winter Savory (Satureja montana L.): Antioxidant Assessment and Chemometric Interpretation
Source: Plants (Basel). 2025 Jul 17;14(14):2214. doi: 10.3390/plants14142214 (PMC12300982; doi:10.3390/plants14142214)
Supplement: Supplementary file 1 [file plants-14-02214-s001.zip › plants-3720582-supplementary.pdf]

Supplementary material

Table S1. TPC and TFC of wintery savory extracts obtained by HVED at different conditions.

Extraction conditions	TPC (mg GAE/g DW)	TFC (mg CE/g DW)
40 Hz, 1 min	39.88±4.44^{ce}	16.30±0.36^{d}
40 Hz, 5 min	30.85±0.84^{g}	13.34±0.24^{f}
40 Hz, 15 min	34.79±1.88^{efg}	14.49±0.22^{ef}
40 Hz, 30 min	43.06±3.23^{cd}	17.64±0.22^{c}
40 Hz, 45 min	46.35±4.44^{bc}	18.24±0.31^{c}
70 Hz, 1 min	26.33±1.12^{h}	10.93±0.21^{g}
70 Hz, 5 min	32.46±0.62^{fgh}	13.03±0.16^{f}
70 Hz, 15 min	34.22±0.87^{efg}	13.01±0.13^{f}
70 Hz, 30 min	41.60±0.30^{cd}	16.81±0.94^{cd}
70 Hz, 45 min	52.04±3.57^{a}	24.17±1.43^{ab}
100 Hz, 1 min	38.49±2.85^{def}	15.47±0.25^{de}
100 Hz, 5 min	34.90±1.02^{fg}	14.44±0.14^{ef}
100 Hz, 15 min	40.92±1.65^{ce}	16.63±0.10^{cd}
100 Hz, 30 min	58.74±2.08^{a}	22.59±0.16^{b}
100 Hz, 45 min	50.47±3.09^{ab}	24.35±0.37^{a}

Different letters within the column represents significant differences among the samples for each response, determined at a significance level of $p < 0.05$.

Table S2. Antioxidant activities (mg/mL) of wintery savory extracts obtained by HVED at different conditions: DPPH, ABTS and FRAP.

Extraction conditions	DPPH (IC_{50})	ABTS (IC_{50})	FRAP (EC_{50})
40 Hz, 1 min	0.0369±0.0004^{bc}	0.0348±0.0005^{cde}	0.1188±0.0008^{e}
40 Hz, 5 min	0.0406±0.0009^{b}	0.0278±0.0016^{e}	0.1282±0.0010^{e}
40 Hz, 15 min	0.0401±0.0030^{b}	0.0336±0.0003^{cde}	0.1632±0.0027^{bc}
40 Hz, 30 min	0.0348±0.0011^{c}	0.0243±0.0015^{e}	0.1324±0.0009^{de}
40 Hz, 45 min	0.0335±0.0014^{c}	0.0139±0.0041^{f}	0.1809±0.0814^{de}
70 Hz, 1 min	0.0564±0.0044^{a}	0.0636±0.0104^{a}	0.2071±0.0063^{a}
70 Hz, 5 min	0.0505±0.0043^{a}	0.0437±0.0012^{bc}	0.1841±0.0057^{ab}
70 Hz, 15 min	0.0539±0.0023^{a}	0.0464±0.0033^{b}	0.1855±0.0011^{ab}
70 Hz, 30 min	0.0339±0.0004^{c}	0.0413±0.0018^{bcd}	0.1593±0.0278^{bcd}
70 Hz, 45 min	0.0211±0.0005^{d}	0.0327±0.0001^{de}	0.0661±0.0329^{f}
100 Hz, 1 min	0.0376±0.0015^{bc}	0.0382±0.0012^{bcde}	0.1291±0.0031^{e}
100 Hz, 5 min	0.0369±0.0009^{bc}	0.0489±0.0015^{b}	0.1550±0.0131^{bcd}
100 Hz, 15 min	0.0419±0.0010^{b}	0.0445±0.0017^{bc}	0.1444±0.0037^{cde}
100 Hz, 30 min	0.0240±0.0003^{d}	0.0347±0.0007^{cde}	0.1274±0.0013^{e}
100 Hz, 45 min	0.0201±0.0002^{d}	0.0340±0.0069^{bcde}	0.1282±0.0027^{e}

Different letters within the column represents significant differences among the samples for each response, determined at a significance level of $p < 0.05$.

Table S3. The sum of quantified individual phenolic compounds in winter savory extracts obtained by HVED at different conditions.

Sample		Σ Phenolics (μg/g DW)
**40 Hz**	1 min	206.95±12.37[h]
	5 min	340.05±23.55[g]
	15 min	382.45±21.14[g]
	30 min	916.80±5.52[e]
	45 min	1226.20±49.50[d]
**70 Hz**	1 min	313.15±14.35[g]
	5 min	731.80±16.69[f]
	15 min	884.80±46.53[e]
	30 min	701.65±7.85[f]
	45 min	2127.75±21.01[b]
**100 Hz**	1 min	1002.05±3.18[de]
	5 min	1118.30±100.97[d]
	15 min	1248.65±68.09[d]
	30 min	1608.95±85.35[c]
	45 min	2657.50±137.04[a]

Different letters within the column represents significant differences among the samples, determined at a significance level of $p < 0.05$.
